# A Predictive Model of a Driver’s Target Trajectory Based on Estimated Driving Behaviors

**DOI:** 10.3390/s23031405

**Published:** 2023-01-26

**Authors:** Zhanhong Yan, Bo Yang, Zheng Wang, Kimihiko Nakano

**Affiliations:** The Institute of Industrial Science, The University of Tokyo, Tokyo 153-8505, Japan

**Keywords:** advanced driver assistance system, driver behavior, deep neural network

## Abstract

With the development of automated driving, inferring a driver’s behavior can be a key element for designing an Advanced Driver Assistance System (ADAS). Current research is focused on describing and predicting a driver’s behaviors as labels, e.g., lane shifting, lane keeping, etc., during driving. In our work, we consider that predicting a driver’s behavior can be described as predicting a trajectory the driver may follow in the near future. The target trajectory can be calculated through certain polynomial functions. Via the data set collected by a Driving Simulator experiment covering nine volunteers, we proposed a model based on a deep learning network which is capable of predicting the corresponding coefficients of polynomial functions and then generating the trajectories in the next few seconds. The results also discussed and analyzed some possible factors affecting the prediction error. In conclusion, the model proved to be effective in predicting the target trajectory of a driver.

## 1. Introduction

Driving is becoming a complex activity with the increasing number of road vehicles. According to a report from the World Health Organization [1,2], every year over one million people are injured or killed due to traffic accidents. Some researchers noticed that many crashes occur during merging or changing lanes, and human errors were one of the major reasons [3,4]. As a result, the Advanced Driver Assistance System (ADAS) has become a promising system for reducing driving workload and helping drivers deal with complex situations. One classic function of an ADAS is the Lane-Keeping Assist System (LKAS), which can prevent unintentional lane departure by alert or vibration [5,6]. Some researchers also explored its application in assisting drivers to keep or change lanes [7,8].

ADASs show the potential of improving driving safety and efficiency. Predicting a driver’s behavior will help the ADAS to serve the driver more efficiently. Supposing that the LKAS is mistakenly activated when a driver tries to change lanes, he/she will feel interference from the ADAS [7]. Previous research also suggests that early detection of potential human errors can help the system to take corrective action such as braking, therefore reduce collision possibility [9]. In general, a driver’s behaviors can be classified into labels, such as changing or keeping lanes, merging, turning left, etc. Researchers have made a lot of effort in improving behavior prediction accuracy. Kumar et al. proposed a method based on the Support Vector Machine (SVM) and the Bayesian filter [10]. The model comprised vehicle dynamic input to detect the intention of lane shifting and predict probabilities of different behaviors as results. The data set was established on road experiments, but the selected features for prediction did not include any driver’s states such as eye or head movements. Kim et al. considered a preprocessing method based on road conditions and vehicle kinematic data to detect a driver’s intention [11]. Their data set was collected via a Driving Simulator (DS) experiment. Kim also explored different combinations in influencing prediction accuracy. Lethaus et al. proved that the gaze movements of a driver alone can be used for predicting behaviors [4]. They divided a driver’s vision into five areas: windshield, left and right window, rear-view mirror and speed indicator. By estimating the time that a driver’s gaze stayed in a certain place, they compared the performance of Neural Network, Bayesian Networks and Naïve Bayes Classifiers for intention prediction. Moreover, in [12], Dang et al. defined the behavior classification problem as a regression problem of when the ego vehicle will cross the borderline. They proved that a Deep-Neural-Network-based model was capable of predicting the time of crossing.

The researchers used yaw angle, lane departure distance, etc., to distinguish driving behaviors. As an example, a lane-changing behavior can be defined as when the vehicle crossed a lane boundary. However, there are in fact no clear boundaries for differentiating such behaviors. For a driver, driving behaviors such as changing or keeping lanes are tracking a continuous trajectory, and the labels are manually tagged without clear rules. A more ideal way is predicting a driver’s target trajectory in the upcoming few seconds. The previous research suggests in comparison with giving a classification label, a predicted target trajectory could be more helpful for ADAS [13,14,15]. A target trajectory contains more information than a simple label and can help ADASs in a more efficient way.

**Figure 1:**
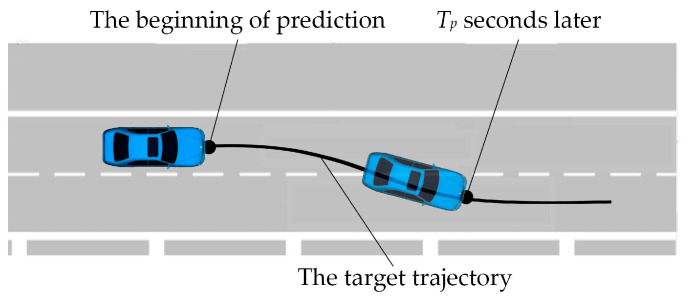
Prediction of a target trajectory (*T_p_* seconds later).

To be more specific, a target trajectory prediction problem can be described as shown in Figure 1, which asks the following question: at the current time *t*_0_, what is the trajectory that the driver may follow in the next *T_p_* seconds?

In [16], Khakzar et al. proposed a method of prediction derived from the Second Strategic Highway Research Program (SHRP2) Naturalistic Driving Study (NDS) [17]. The researchers proposed a dual learning model and risk map for vehicle trajectory prediction. Khakzar et al. built a Deep Neural Network (DNN) to predict the behaviors in the upcoming future. They also analyzed the factors affecting prediction error such as drivers’ profiles. The conclusion suggested that self-reported questionnaires showed no significant difference in driving performance information. Liu used a kinematic model to predict the trajectories of the preceding vehicle [18]. They described the trajectory by calculating the corresponding coefficients of a cubic polynomial function. Other kinematic models such as Constant Turn Rate or Constant Velocity were also discussed and utilized for the prediction [19]. On the other hand, in [20], Hu et al. established a Gaussian mixture model based on a DNN to predict the probability distribution areas where the vehicle is occupied. Some researchers pointed out that such DNN-based methods consider the interactions vehicle-to-vehicle and environment-to-vehicle effectively. The fusion of information can help to improve behavior prediction accuracy [21,22]. 

Clearly, great achievements have been made in understanding and predicting the driver’s behavior. Previous research suggested that inferring the trajectories of a vehicle could be helpful for designing a more powerful ADAS. In this work, we proposed a method by considering a driver’s observing behavior, kinematic parameters and environmental factors. By considering those factors, our model can predict a driver’s target trajectory. We also compared the different combinations of features for reducing prediction error. The finding of effective features can be used for designing an accurate prediction model. The testing results suggest that our model can give an accurate prediction of the target trajectory. 

This paper is organized as follows. Section 2 tells the methodology of describing and predicting the trajectory. Section 3 describes the Driving Simulator experiment, including the scenario, conditions, apparatus, participants and procedure. Section 4 provides the results of the experiment, and is followed by Section 5, where the performance of the system is discussed. Finally, the conclusion is presented in Section 6.

## 2. Trajectory Based on Polynomial Functions

As described in Section 1, if the current time is *t*_0_, our target is to predict the future positions of the ego vehicle in the next *T_p_* seconds, as shown in Figure 1.

Polynomial functions have been proven effective in predicting and planning a smooth local trajectory [23,24]. Moreover, higher polynomials can be used to describe motions in lateral and longitudinal directions independently.

According to [23,25], the lateral motion is performed as

(1) $$y_{r}\left(t\right)=b_{0}+b_{1}t+b_{2}t^{2}+b_{3}t^{3}+b_{4}t^{4}+b_{5}t^{5}$$

For the lateral motion, we assume the initial state is ${[y}_{r0}\;\dot{y_{r0}}\;\ddot{y_{r0}}]$, representing the lateral position, velocity, and acceleration at the beginning. Similarly, the final state is given as ${[y}_{rf}\;\dot{y_{rf}}\;\ddot{y_{rf}}$].

The longitudinal motion is

(2) $$x_{r}\left(t\right)=a_{0}+a_{1}t+a_{2}t^{2}+a_{3}t^{3}+a_{4}t^{4}$$

The initial state is defined as $\left[x_{r0}\;\dot{x_{r0}}\;\ddot{x_{r0}}\right]$, representing the longitudinal position, velocity, and acceleration at the beginning. The final state is given as $\left[\dot{x_{rf}}\;\ddot{x_{rf}}\right]$, since the final longitudinal position is unknown a priori. 

Further, $x_{r0}$ can be obtained by substituting *t* = 0 into (2):

(3) $$x_{r0}=a_{0}+a_{1}•0+a_{2}•0+a_{3}•0+a_{4}•0$$

Taking the derivative of (2), $\dot{x_{r0}}$ can be obtained by substituting *t* = 0 into (4):

(4) $$\dot{x_{r}\left(t\right)}=a_{1}+2a_{2}t+3a_{3}t^{2}+4a_{4}t^{3}$$

(5) $$\dot{x_{r0}}=a_{1}+2a_{2}•0+3a_{3}•0+4a_{4}•0$$

Similarly, we have

(6) $$\ddot{x_{r0}}=2a_{2}+3a_{3}•0+4a_{4}•0$$

Substituting *t* = *T_p_*, the prediction horizon as the final state, we have

(7) $$\dot{x_{rf}}=a_{1}+2a_{2}T_{p}+3a_{3}T_{p}{}^{2}+4a_{4}T_{p}{}^{3}$$

(8) $$\ddot{x_{rf}}=2a_{2}+6a_{3}T_{p}+12a_{4}T_{p}{}^{2}$$

For the lateral direction, we can obtain ${[y}_{r0}\;\dot{y_{r0}}\;\ddot{y_{r0}}]$ and ${[y}_{rf}\;\dot{y_{rf}}\;\ddot{y_{rf}}$] via the same way.

If the coefficients in (1) and (2) are known, the trajectories in lateral and longitudinal directions can be defined by time *t*. With the initial and final states, the coefficients in the polynomials can be obtained by solving the following equations:

(9) $${\left[a_{0}\;\;a_{1}\;\;a_{2}\;\;a_{3}\;\;a_{4}\right]}^{T}=A^{-1}X_{r}$$

(10) $${\left[b_{0}\;\;b_{1}\;\;b_{2}\;\;b_{3}\;\;b_{4}\;\;b_{5}\right]}^{T}=B^{-1}Y_{r}$$

where the matrix ***A*** is given by (3), (5), (6), (7) and (8) (same for matrix ***B***). ***X_r_*** and ***Y_r_*** are the initial and final states, as follows:

(11) $$A=\left[\begin{array}{ccccc} 1 & 0 & 0 & 0 & 0\\ 0 & 1 & 0 & 0 & 0\\ 0 & 0 & 2 & 0 & 0\\ 0 & 1 & 2T_{p} & 3T_{p}{}^{2} & 4T_{p}{}^{3}\\ 0 & 0 & 2 & 6T_{p} & 12T_{p}{}^{2} \end{array}\right]$$

(12) $$B=\left[\begin{array}{cccccc} 1 & 0 & 0 & 0 & 0 & 0\\ 0 & 1 & 0 & 0 & 0 & 0\\ 0 & 0 & 2 & 0 & 0 & 0\\ 1 & T_{p} & T_{p}{}^{2} & T_{p}{}^{3} & T_{p}{}^{4} & T_{p}{}^{5}\\ 0 & 1 & 2T_{p} & 3T_{p}{}^{2} & 4T_{p}{}^{3} & 5T_{p}{}^{4}\\ 0 & 0 & 2 & 6T_{p} & 12T_{p}{}^{2} & 20T_{p}{}^{3} \end{array}\right]$$

(13) $$X_{r}=\left[\begin{array}{c} x_{r0}\\ \dot{x_{r0}}\\ \ddot{x_{r0}}\\ \dot{x_{rf}}\\ \ddot{x_{rf}} \end{array}\right]$$

(14) $$Y_{r}=\left[\begin{array}{c} y_{r0}\\ \dot{y_{r0}}\\ \ddot{y_{r0}}\\ y_{rf}\\ \dot{y_{rf}}\\ \ddot{y_{rf}} \end{array}\right]$$

Therefore, we have:

(15) $$\{\begin{array}{c} \begin{array}{c} a_{0}=x_{r0}\\ a_{1}=\dot{x_{r0}}\\ a_{2}=\ddot{x_{r0}}/2\\ a_{3}=-\frac{3\dot{x_{r0}}-3\dot{x_{rf}}+2T_{p}\ddot{x_{r0}}+T_{p}\ddot{x_{rf}}}{3T_{p}{}^{2}}\\ a_{4}=\frac{2\dot{x_{r0}}-2\dot{x_{rf}}+T_{p}\ddot{x_{r0}}+T_{p}\ddot{x_{rf}}}{4T_{p}{}^{3}} \end{array} \end{array}$$

(16) $$\{\begin{array}{c} \begin{array}{c} b_{0}=y_{r0}\\ b_{1}=\dot{y_{r0}}\\ b_{2}=\frac{\ddot{y_{r0}}}{2}\\ b_{3}=\frac{-\left(20y_{r0}-20y_{rf}+12T_{p}\dot{y_{r0}}+8T_{p}\dot{y_{rf}}+3T_{p}{}^{2}\ddot{y_{r0}}-T_{p}{}^{2}\ddot{y_{rf}}\right)}{2T_{p}{}^{3}}\\ b_{4}=\frac{30y_{r0}-30y_{rf}+16T_{p}\dot{y_{r0}}+14T_{p}\dot{y_{rf}}+3T_{p}{}^{2}\ddot{y_{r0}}-2T_{p}{}^{2}\ddot{y_{rf}}}{2T_{p}{}^{4}}\\ b_{5}=\frac{-\left(12y_{r0}-12y_{rf}+6T_{p}\dot{y_{r0}}+6T_{p}\dot{y_{rf}}+T_{p}{}^{2}\ddot{y_{r0}}-T_{p}{}^{2}\ddot{y_{rf}}\right)}{2T_{p}{}^{5}} \end{array} \end{array}$$

The coefficients in (1) and (2) can be obtained with (15) and (16). Since the initial states are known, the prediction of a trajectory depends on the final states, $\dot{x_{rf}},\;\ddot{x_{rf}}$ and $y_{rf},\;\dot{y_{rf}},\;\ddot{y_{rf}}$. Therefore, the target turns into predicting the five variables as the final states.

Here, we re-form the problem of prediction as the following description:

For time moment *t*, we assume the data collected from sensors are ***S_t_***. Assuming the current time is *t*_0_, all the data collected in the past *k* time step up to *t*_0_ form a matrix, $S_{t_{0}}{=(s}_{t_{0}-k+1}{,s}_{t_{0}-k+2}{,\ldots ,s}_{t_{0}-1}{,s}_{t_{0}})$. The problem changes into finding a model that gives the prediction of the final states in the longitudinal and lateral direction in *T_p_* seconds later.

## 3. Methodology

With a clear definition of the issue, the key issue turns into building a regression model. The Recurring Neural Network (RNN) is proven to be an efficient tool for such tasks, particularly for a problem that is difficult to describe in a simple mathematical function. Meanwhile, as a popular RNN, the Gated-Recurrent-Unit (GRU) network has received a great deal of attention since its proposal in 2014 [26]. It is a powerful tool in time-series-based classification and regression problems. In addition, GRU has a simplified architecture, but shows similar performance in lots of tasks [26] in comparison with another widely used Recurrent Neural Network, Long-Short-Term-Memory (LSTM) [27]. 

The RNN-based deep learning method is widely used for solving such regression problems. It requires data sets for training and testing. Therefore, we conducted a Driving Simulator (DS) experiment for collecting data.

### 3.1. Data Set

Nine participants (including seven men and two women) were invited to participate in the experiment. Volunteers ranged in age between 19 and 26 years (mean age = 22.8, standard deviation = 2.4). All participants held a Japanese driver’s license (mean driving years = 2.6 years, standard deviation = 1.9 years). All participants received payment for their participation. The experiment was endorsed by the University of Tokyo’s Office for Life Science Research Ethic and Safety.

**Figure 2:**
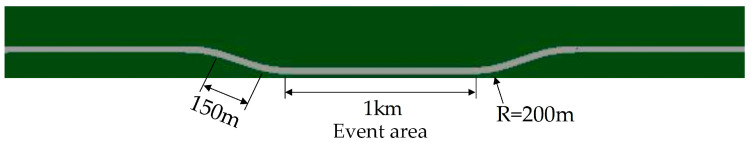
Segment for lane-changing event in DS environment.

Each participant drove on a highway in a simulator environment (as shown in Figure 2). Participants were free to steer the car and were suggested to drive under a speed limitation (60 km/h). The experiment scenario included several segments, in which a leading vehicle whose speed was limited to slower than the ego vehicle then triggered a lane change event to collect driving trajectories. For each event, the leading vehicle would decelerate at a certain rate which was randomly selected from −1 m/s^2^ to −3 m/s^2^ until its speed was slower than the ego vehicle with at least 10 km/h difference.

**Figure 3:**
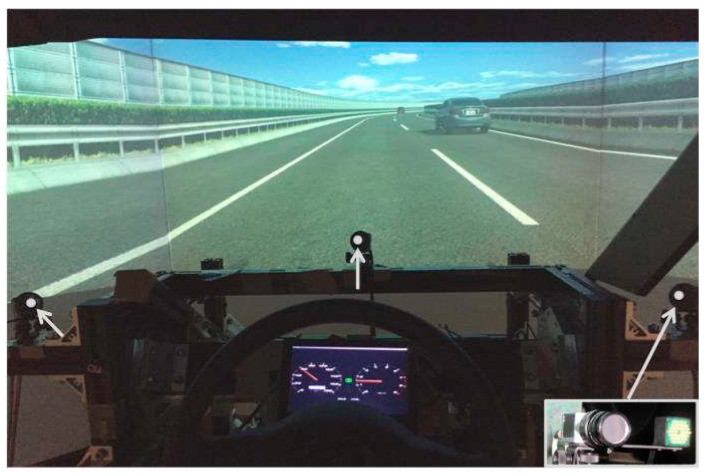
Smart Eye Pro eye-tracking system and DS image. The eye-tracking device relies on infrared cameras (indicated in the photo) to track the movement of the head and eyes.

The details of sensor data $S_{t_{0}}$ for prediction are shown in Table 1. A gaze-tracking system was used to measure the gaze and head movements [28]. The gaze-tracking system (in Figure 3) did not create physical burdens on the participants. All the drivers drove in a natural state and were told to follow the basic traffic rules.

Therefore, $S_{t_{0}}$ is

$$S_{t_{0}}=\left[\begin{array}{c} \begin{array}{ccccc} {Head}_{x,t_{0}-k+1} & Gaze_{y,t_{0}-k+1} & \ldots & d_{t_{0}-k+1} & \psi_{t_{0}-k+1}\\ {Head}_{x,t_{0}-k+2} & Gaze_{y,t_{0}-k+2} & \ldots & d_{t_{0}-k+2} & \psi_{t_{0}-k+2}\\ \ldots & \ldots & \ddots & \ldots & \ldots \\ {Head}_{x,t_{0}} & Gaze_{y,t_{0}} & \ldots & d_{t_{0}} & \psi_{t_{0}} \end{array} \end{array}\right]$$

### 3.2. Labeling

**Figure 4:**
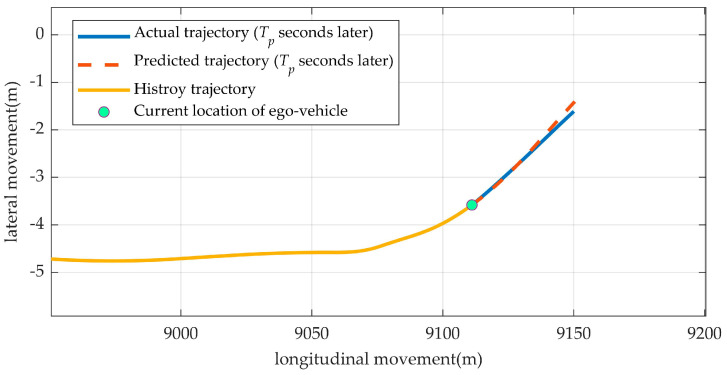
An example of a predicted target trajectory (in *T_p_* later).

The prediction model outputs a vector [$\dot{x_{rf}},\;\ddot{x_{rf}},y_{rf},\dot{y_{rf}},\;\ddot{y_{rf}}$], as mentioned in Section 2. An example of predicting a trajectory is shown in Figure 4. The model predicted [$\dot{x_{rf}},\;\ddot{x_{rf}},y_{rf},\dot{y_{rf}},\;\ddot{y_{rf}}$] in the next *T_p_* seconds then obtain the coefficients in polynomials from (15) and (16). By having the coefficients, the trajectory can be calculated.

### 3.3. Network Architecture

A short time before the present moment may cover important information to predict future behaviors, because drivers are meant to perform a series of behaviors prior to a maneuverer. As a result, this task requires a method that is able to remember the information at an earlier time and uncover the hidden dependence in the time series. RNN can retain a state and has been proven effective in performing such tasks. However, researchers pointed out that the vanilla RNN had a potential risk of gradient exploding or vanishing. These problems sometime made the vanilla RNN hard to train [29]. GRU and LSTM are two popular extended RNNs [26,27]. In comparison with the vanilla RNN, their modified architectures overcome such disadvantages. Both LSTM and GRU achieve memory function through a gating mechanism, but GRU simplifies the architecture and does not have memory nodes. The performance of the two RNNs is similar for most of the tasks, but GRU usually needs less time to train. In this research, we focused on utilizing GRU to build the model [7].

GRU can decide how much the unit should update its content through two gates and remember information through the unit *hidden state* ${h\;}_{t}^{j}$. To update ${h\;}_{t}^{j}$ (*j* = the *j*-th unit, *t* = time step), the process is as follows:

$$\mathrm{Hidden}\;\mathrm{state}:\;{h\;}_{t}^{j}{=(1-z\;}_{t}^{j}{)h\;}_{t-1}^{j}+z{\;}_{t}^{j}{\tilde{h\;}}_{t}^{j}$$

where ${z\;}_{t}^{j}$ is the *update gate* controlling how much content should be updated. When ${z\;}_{t}^{j}=0$, ${h\;}_{t-1}^{j}$ will be copied to ${h\;}_{t}^{j}$ without loss; ${z\;}_{t}^{j}$ is described as

$$\mathrm{Update}\;\mathrm{gate}:\;z_{t}^{j}{=\sigma (W}_{z}x_{t}{+U}_{z}h_{t-1}{)}^{j}$$

where the *new memory* ${\tilde{h\;}}_{t}^{j}$ summarizes the information from the new entry **x***_t_* and *h_t_*_−1_ (the *hidden state* at time step *t* − 1):

$$\mathrm{New}\;\mathrm{memory}:\;{\tilde{h\;}}_{t}^{j}{=\tanh(W}_{z}x_{t}{+U(r}_{t}⨀h_{t-1}{))}^{j}$$

where ${r\;}_{t}^{j}$ is the *reset gate* deciding the contents to be abandoned from the *hidden state*. The previous hidden state ${h\;}_{t-1}^{j}$ will not pass to the new memory ${\tilde{h}}_{t}^{j}$ if ${r\;}_{t}^{j}=0$, where ${r\;}_{t}^{j}$ is described as

$$\mathrm{Reset}\;\mathrm{gate}:\;r_{t}^{j}{=\sigma (W}_{r}x_{t}{+U}_{r}h_{t-1}{)}^{j}$$

the *hidden state* representing the memory can be updated selectively through the *update gate* and the *reset gate*. *W_r_*, *W_z_*, *U_r_* and *U_z_* are learnable parameters during the network training.

**Figure 5:**
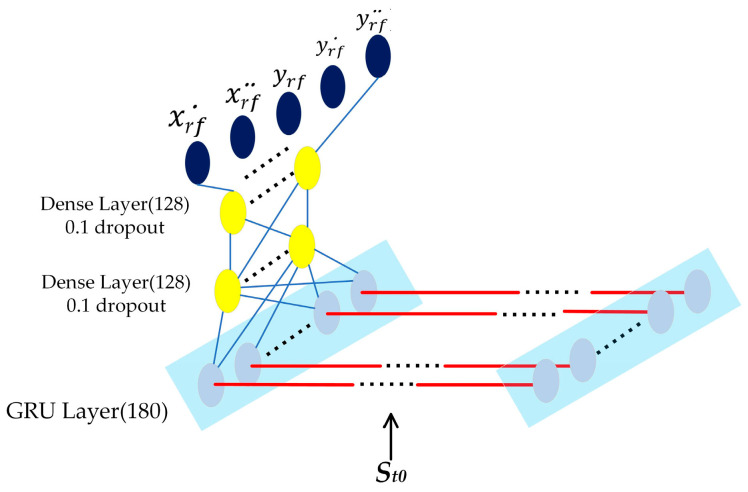
The Neural Network architecture.

In this research, the network begins with a layer of GRU with 180 units and is connected by two dense layers with 128 units. When we validated our method, we found that more recurrent units would cost significantly more time to train but did not improve the accuracy obviously, while reducing the number of network units caused the network to lack the ability to predict. Figure 5 shows the network architecture.

## 4. Results

A data set covering nine participants was collected during the experiment. Each participant drove for about 45 to 60 minutes on a highway. All the participants were free to steer or accelerate while driving and could change lanes whenever they thought necessary. In each trial, the drivers were required to follow the basic traffic rules in Japan. 

The data set covered trajectories during the whole driving process. We collected 23,168 samples in total, including 16,256 samples for training, 4032 samples for validating and 2880 samples for testing. *T_p_* was set as four seconds for our research. Each sample contained an $S_{t_{0}}$ and [$\dot{x_{rf}},\;\ddot{x_{rf}},y_{rf},\dot{y_{rf}},\;\ddot{y_{rf}}$] at corresponding times. The testing samples were from three randomly selected participants. Training samples were shuffled and collected from all participants. We fixed the sampling window as three seconds and the sampling rate = 60 Hz. The time step parameter *k* in $S_{t_{0}}$ was set as 180.

### 4.1. Predicted Trajectories

**Figure 6:**
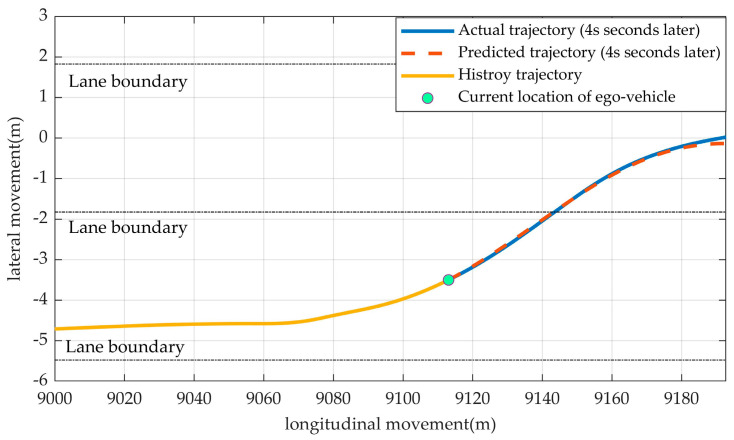
A predicted target trajectory when the driver was changing lanes (from subject 7).

Following the steps in the previous section, we established a model for predicting a driver’s target trajectories four seconds (*T_p_* = 4 s) later, as shown in Figure 6. The trajectory was from subject 7 during lane changing.

Since the predicted trajectory and the actual trajectory were almost aligned, our model could give a prediction of the driver’s target trajectory.

### 4.2. Feature Selection for Prediction

For a driver, he/she intends to check side mirrors to make sure there is a safe environment to perform maneuvers. Observational behaviors can be useful for predicting trajectories. Some researchers pointed out that the gaze and head data performed closely on improving accuracy for driving behavior prediction [3,4]. However, more discussion is still needed to evaluate the roles of gaze and head dynamic data in trajectory prediction performance. Here, we compared the Final Displacement Error (FDE) that was obtained from models trained via different combinations of the features in Table 1. The FDE is given by

(17) $$FDE=\sqrt{{{(x}_{t,i}^{pred}{-x}_{t,i}^{actual})}^{2}}$$

where $x_{t,i}^{pred}$ and $x_{t,i}^{actual}$ are the predicted position and the actual position of a trajectory at time step *t*, respectively, *i* = the *i*-th sample and *t* = *T_p_* = 4 s. FDE suggests the general performance of trajectory prediction. 

Table 2 shows that combination (*d*) has the smallest prediction error in the lateral direction among the four groups. The combination (*a*) without driver states showed the largest error. 

In Table 3, the Kruskal–Wallis tests between each pair of combinations indicated that combination (*d*) did not show a significant difference in comparison with combination (*c*). Combination (*c*), which only included head dynamic data, also significantly reduces the prediction error compared with combination (*a*), while combination (*b*) showed no difference in improving the prediction accuracy.

Meanwhile, for longitudinal direction, the combinations did not show differences in reducing the prediction error. 

### 4.3. Trajectory Prediction Performance

**Figure 7:**
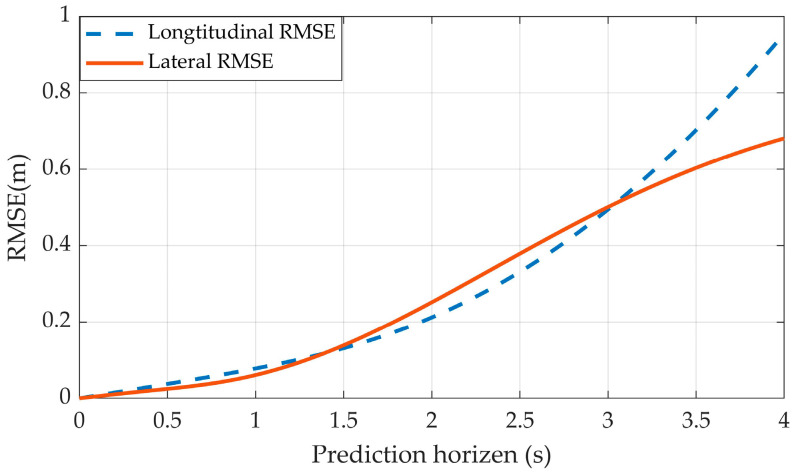
The RMSE in lateral and longitudinal directions. When predicted horizon = 4 s, the prediction reaches max error with 0.68 m (lateral) and 0.95 m (longitudinal).

To further evaluate the prediction performance, we estimated the Root Mean Square Error (RMSE), given by (18) as shown in Figure 7:

(18) $$RMSE=\sqrt{\frac{1}{n}\overset{n}{\underset{i=1}{\sum }}{{(x}_{t,i}^{pred}{-x}_{t,i}^{actual})}^{2}}$$

where $x_{t,i}^{pred}$ and $x_{t,i}^{actual}$ are the predicted position and the actual position of a trajectory at time step *t*, respectively, *n* = the number of samples and *i* = the *i*-th sample. 

Figure 7 depicts the lateral and longitudinal RMSE of the prediction model up to four seconds. It can be seen that with the prediction horizon increasing, the prediction error also increases in both directions. 

For each sample, we also estimated its Final Displacement Difference (FDD) in both directions. The FDD is given by

(19) $$FDD=x_{t,i}^{pred}{-x}_{t,i}^{actual}$$

where $x_{t,i}^{pred}$, $x_{t,i}^{actual}$, *i* and *t* are the same as in (17) and *t* = *T_p_* = 4 s.

**Figure 8:**
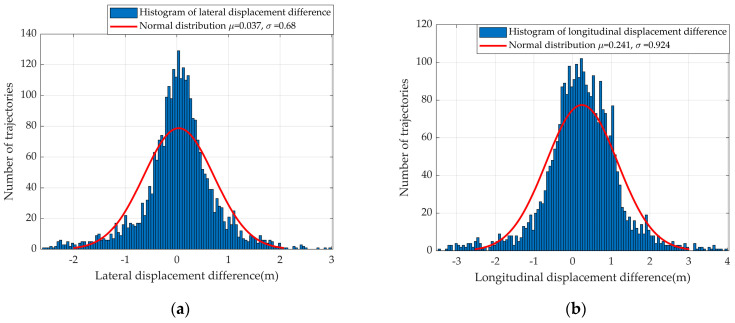
(**a**) The histogram of lateral displacement difference with fitted normal distribution. (**b**) The histogram of longitudinal displacement difference with fitted normal distribution.

Figure 8 suggests the prediction displacement distribution. For lateral movement, the FDD followed a normal distribution with μ = 0.037 and σ = 0.68, as shown in Figure 8a. For longitudinal movement, the FDD followed a normal distribution with μ = 0.241 and σ = 0.924, as shown in Figure 8b. 

**Figure 9:**
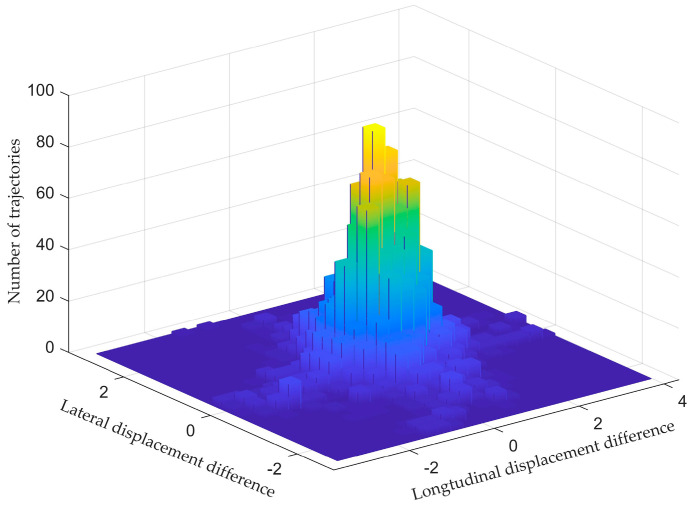
The histogram of lateral and longitudinal displacement difference.

Figure 9 describes the displacement distribution in both lateral and longitudinal directions. It can be seen that the displacement difference distributes around (0, 0).

### 4.4. Comparison of Different Neural Networks

**Figure 10:**
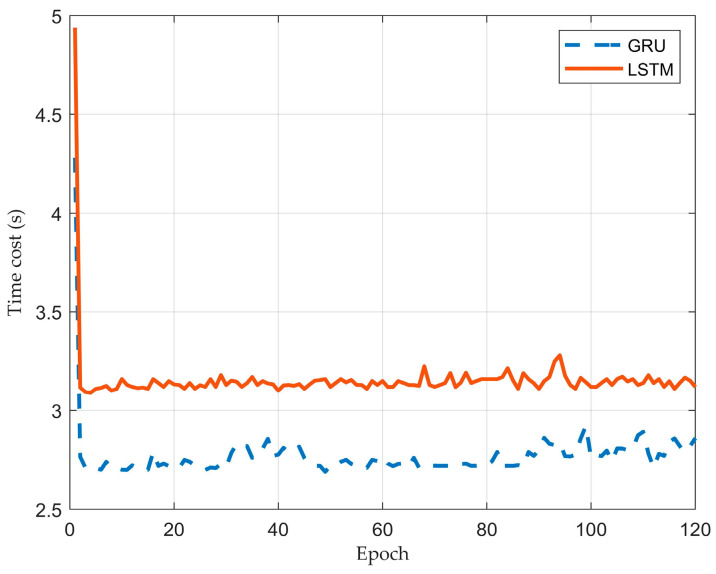
The time costs of LSTM and GRU.

LSTM and GRU have similar architectures, but GRU has fewer parameters, which makes GRU less prone to over-fitting and easier to train [7]. Tuning the LSTM network will take a longer time and more epochs than GRU by the same optimizer [29,30]. Here, we trained two models with the same architecture as Figure 5 and made a comparison of their time costs under the same environment. All other training parameters were also kept the same. The time costs are shown in Figure 10 and Table 4.

The training was run on an nVIDIA GTX 1070 with Cudnn acceleration and lasted 120 epochs. It can be seen from Figure 10 and Table 4 that GRU spent significantly less time than LSTM for each training epoch. In fact, in order to tune LSTM and GRU to reach almost the same performance, LSTM needs about 10%~15% more time than GRU. 

Meanwhile, LSTM did not significantly reduce the prediction error. Therefore, for this task, GRU was proved more suitable than LSTM. 

## 5. Discussion

**Figure 11:**
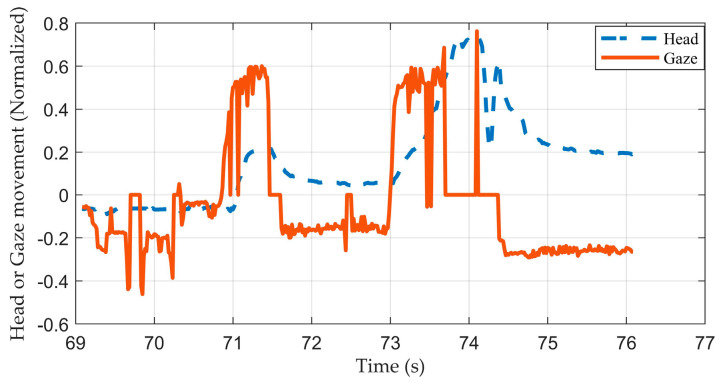
The head movement and gaze movement of subject 7 before changing lanes (occurred at about time = 75 s).

The purpose of this research is to propose a method of predicting a driver’s target trajectory in the upcoming future. We compared the combinations of features to select the most effective set for reducing the prediction error. Combination (*d*) (with *gaze* and *head*) showed the smallest error among the four combinations. Taking combination (*a*) as the baseline, it can be seen from Table 2 that combination (*b*) (only including *gaze*) did not improve the prediction accuracy. Combinations (*d*) and (*c*) (only including *head*) also indicated no difference. These results suggested that *gaze* might have no effect in improving the accuracy. It is possible that *gaze* is not robust enough for monitoring a driver’s behavior, as shown in Figure 11.

It can be seen that when time went to 73.7 to 74.5 s, *Gaze* suddenly jumped to zero. This occurred when our eye-tracking device was unable to capture the gaze movement and it happened a few times. Instead, *head* remained consistent. A driver may peek at mirrors and move their head while driving. Since the cameras were fixed, it was likely that the cameras failed to track the driver’s pupils when he/she moved too fast. These results correspond with other research [3,7], indicating that sometimes gaze movements are not stable enough for estimating a driver’s behavior accurately. In addition, in a few situations, the system may give a prediction with relatively big errors. These may also result from the noise of head or gaze movement. Such conclusions suggest that for an accurate trajectory prediction model, head movement data are essential, but gaze movement, due to its unreliability, may not affect the prediction results significantly. 

We evaluated the RMSE and FDD of predicting a trajectory across the testing set, and the RMSE estimated based on our method was smaller than the prediction method in [2]. For longitudinal movement, the FDD distribution followed a normal distribution with μ = 0.241 and σ = 0.924, which also suggests less error than the method in [2]. However, for lateral movement, the FDD followed a normal distribution, which suggests that our method can predict a driver’s target trajectory in both directions accurately. 

We compared the performance of LSTM and GRU. LSTM and GRU performed closely for our task, but training GRU usually takes less time, which makes it easier to use and tune.

## 6. Conclusions

This research explores a way to predict a driver’s target trajectory by describing a trajectory via polynomials on lateral and longitudinal directions separately. Through a DS experiment, we collected a data set covering drivers, environment and driver states. A model based on GRU was established, trained and validated. The results suggested that a robust prediction model mainly relied on head movement monitoring. In the Discussion, the reasons for the unreliability of gaze movement were discussed and analyzed. It was because that tracking gaze movement accurately could be hard for the current device. Our method has proved to be effective in predicting a driver’s future trajectory up to four seconds. The GRU showed to be more effective in saving training time without losing prediction accuracy. 

This prediction system can be applied to an ADAS to help the system to better cooperate with a driver. The future plan needs to consider recruiting more volunteers to enlarge the data set. 

## Figures and Tables

**Table 1:** Details of monitoring data.

Data		Description
Driver state	Head movement	*Head*, left/right rotation of the head, the angle in the horizontal direction
	Gaze movement	*Gaze*, the gaze movement measured in the horizontal direction, normalized to [−1, 1]
Vehicle state	Acceleration	*a_x_*, *a_y_* Acceleration in the horizontal and vertical direction
	Velocity	*v_x_*, *v_y_* Velocity in the horizontal and vertical direction
	Steering wheel angle	SWA
Environment state	Lateral distance	*d*, the distance to the adjacent lane
	Yaw angle	*ψ*, the angle between the vehicle’s longitudinal axis and the lane

**Table 2:** Lateral mean FDE of different combinations of features.

Combination		Lateral Mean FDE (m)
(*a*)	Environment + vehicle	0.5789
(*b*)	Environment + vehicle + *gaze*	0.5674
(*c*)	Environment + vehicle + *head*	0.5228
(*d*)	Environment + vehicle + *head* + *gaze*	**0.4835**

The mean FDE = $\frac{1}{n}\sum_{i=1}^{n}\sqrt{{{(x}_{t,i}^{pred}{-x}_{t,i}^{actual})}^{2}}$, where *n* = the number of samples.

**Table 3:** Kruskal–Wallis test of lateral FDE of different combinations of features.

	(*a*)	(*b*)	(*c*)	(*d*)
(*a*)	-	-	-	-
(*b*)	0.707	-	-	-
(*c*)	0.000 ***	0.000 ***	-	-
(*d*)	0.000 ***	0.000 ***	0.888	-

***: *p* < 0.001.

**Table 4:** The time costs of LSTM and GRU.

RNN	Mean Time Cost of Each Epoch (s)
GRU	**2.77** ***
LSTM	3.16

***: *p* < 0.001. The Kruskal–Wallis test result between GRU and LSTM.

## Data Availability

Not applicable.
